# Cellular therapies for treating pain associated with spinal cord injury

**DOI:** 10.1186/1479-5876-10-37

**Published:** 2012-03-06

**Authors:** Lawrence Leung

**Affiliations:** 1 Centre of Neurosciences Study, Queen's University, 18 Stuart Street, Kingston, ON K7L 3N6, Canada; 2 Centre of Studies in Primary Care, Queen's University, 220 Bagot Street, Kingston, ON K7L 5E9, Canada; 3 Department of Family Medicine, Queen's University, 220 Bagot Street, Kingston, ON K7L 5E9, Canada

**Keywords:** Cellular therapies, Spinal cord injury, Pain

## Abstract

Spinal cord injury leads to immense disability and loss of quality of life in human with no satisfactory clinical cure. Cell-based or cell-related therapies have emerged as promising therapeutic potentials both in regeneration of spinal cord and mitigation of neuropathic pain due to spinal cord injury. This article reviews the various options and their latest developments with an update on their therapeutic potentials and clinical trialing.

## Spinal cord injury-demography and economic impact

Causes of spinal cord injury (SCI) include falls, motor vehicle accidents, community violence, sports injury and work-related injuries. Annual incidence rate of SCI ranges from 15 to 40 per million [1] with an average age of onset at under 30. There is male sex predominance over female of up to 5:1, with cervical and thoracic regions being the commonest region of trauma [2-5]. Depending on the severity and level(s) of lesion, spinal cord injury leads to a combination of loss of sensory, motor and autonomic functions, translating to clinical scenarios of paraplegia, tetraplegia, aphagia, incontinence and neuropathic pain. This plethora of sequelae leads to catastrophic loss of quality of life of these young and otherwise healthy patients. Economically it also translates to immense economic costs due to loss of work productivity and the demand of life-long supportive care. It has been estimated that annual costs of health care (including hospitalisation and rehabilitation) for an average patient with spinal cord injury range from US$21,450 in Veterans Health Facilities [6] to US$88,585 in a community base setting [7].

## Neuropathic pain after spinal cord injury

About 65-85% of patients will suffer from pain after spinal cord injury and amongst them, 1/3 will have severe pain [8]. Those who experience pain for longer than 6 months are likely to continue for the next 3 to 5 years [9], with a propensity to worsen over time with other associated symptoms like fatigue, weakness and memory loss [10]. The type of pain experienced after spinal cord injury can be classified as neuropathic, musculoskeletal, visceral and others [11]. In a longitudinal sample of 100 patients followed up to 26 weeks after traumatic spinal cord injury, 40% of them reported musculoskeletal pain, 36% reported neuropathic pain at the level of lesion and 16% reported neuropathic pain below level of lesion [12]. Neuropathic pain is more common with incomplete lesions of the cord and is more often associated with cervical as compared to other levels of injury [12]. Like other types of neuropathic pain, pain due to spinal cord injury remains as a major challenge in pain management and so far the commonest therapy is with opioids, albeit a 32% long term efficacy [13].

### Cellular and molecular basis for neuropathic pain due to spinal cord injury

**Figure 1:**
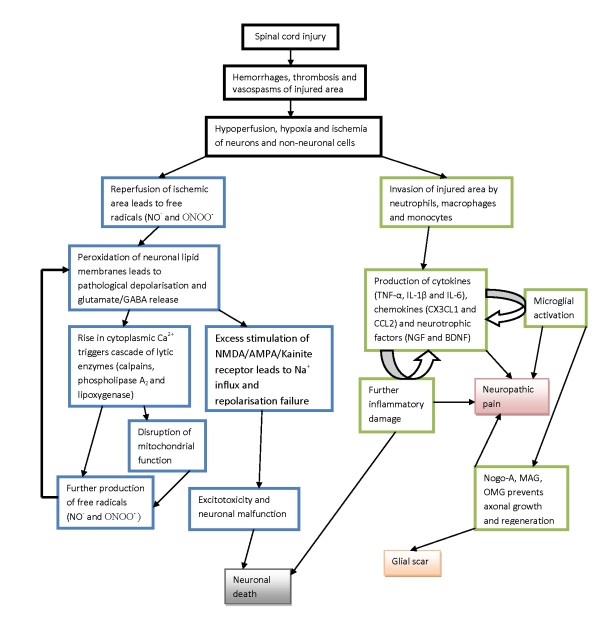
**A simplified flowchart of pathological events after spinal cord injury leading to neuronal death, glial scar and neuropathic pain**.

A typical non-transection injury of the spinal cord results in various degrees of contusion and compression, causing mechanical disruption of microvascular structures resulting in hemorrhages, intravascular thrombosis and vasospasm. This subsequently leads to local hypoperfusion, hypoxia and ischemic damage. Paradoxically, a period of reperfusion of the ischemic tissue occurs when vasospastic blood vessels relax, and this produces free-radicals, notably peroxynitrite (ONOO^-^), which progressively oxidize fatty acids in the cellular membranes (lipid peroxidation). This in turn causes pathological membrane depolarisation and massive release of glutamate from the injured axons, which subsequently over-stimulates post-synaptic N-Methyl-D-aspartic acid (NMDA) and α-amino-3-hydroxy-5-methyl-4-isoxazolepropionic acid (AMPA) receptors. As a result, cytoplasmic calcium inside the injured axons rises abruptly due to influx from extracellular compartment and also release from intracellular stores [14], triggering off a cascade of calcium-dependent processes like activation of lytic enzymes (calpains, phospholipase A_2 _and lipoxygenase), disruption of mitochondrial function and even more free radicals generation (nitric oxide and peroxynitrite)[15], culminating in apoptosis and final death of the axons. Excessive stimulation of NMDA/AMPA/kainite receptors by glutamate also leads to a similar influx of sodium ions into the intracellular compartment of the axons, causing repolarisation failure and loss of axonal function. Outside the injured axons, an inflammatory cascade commences with invasion of neutrophils and monocytes/macrophages which secret an array of immuno-active mediators like cytokines (TNF-α, IL-1β and IL-6), chemokines (CX3CL1 and CCL2) and neurotrophic factors (NGF and BDNF) which often contribute to further inflammatory damage and pave the way for neuropathic pain [16,17]. A summary of events which happen after a spinal cord injury can be found in a schematic flowchart in Figure 1. In recent years, significant progress has been made in profiling the intricate roles of these neuroinflammatory mediators and the astroglial system in the pathogenesis of neuropathic pain, and the topic has been extensively reviewed elsewhere [18-21]. Details are beyond the scope of this paper but worth noting is the latest concept of neuropathic pain as a neuro-immune process with its severity and chronicity determined by the net balance of concurrent pro-inflammatory (neurodegenerative) and anti-inflammatory (neuroprotective) mechanisms [22].

## Cell-based strategies for treating neuropathic pain associated with spinal cord injury

### Using primary adrenal medullary/chromaffin tissue or cells

Descending tracts from the periaqueductal gray (PAG), locus coeruleus, parabrachial nucleus, nucleus raphe magnus, reticular formation, anterior pretectal nucleus, thalamus and cerebral cortex are known to modulate afferent nociceptive signals [23] at the spinal cord. This is thought to mediate via an array of neurotransmitters like brain-derived neurotrophic factors (BDNF), 5-hydroxytrytamine (5-HT), noradrenaline, γ-aminobutyric acid (GABA), β-endorphins, enkephalins and galanin. Since initial discovery of the colour reaction of the adrenal medulla to chromate salt in 1865 by Henle [24], the term chromaffin cells refer to those that contain granules or vesicles which retain the chromium particles upon chromaffin reaction. It was not until 1953 that Hillarp *et al. *[25] and Blaschko *et al. *[26] independently reported the findings of catecholamines in the granular portion of abstract of bovine adrenal medullae. Subsequent work in the 1980's revealed a collection of antinociceptive neuropeptides and neurotrophins in these chromaffin cells [27-29] which in theory, can be utilised on their own or with catecholamines [30,31] in a mini-pump logic for analgesic purposes. This cradled the pioneering work of Sagen *et al. *in 1986 who implanted bovine chromaffin cells into the subarachnoid space of lumbar spine of rats and found significant analgesia which was dose-related to the amount of chromaffin cells [32]. They obtained similar findings with allogeneic transplants of adrenal medullary tissue [33], documenting significant increase of met-enkephalin-like immunoreactivity in the spinal cord cerebral spinal fluid (CSF)[34] and prolongation of analgesia with the use of intrathecal enkephalinase [35], substantiating the role of opioid peptides in the analgesic mechanisms. Using similar adrenal medullary allografts transplanted into the subarachnoid space of rat spinal cord, they demonstrated reduction of chronic pain in rats modeled for arthritis [36] and peripheral neuropathy [37]. These promising data propelled the use of cadaveric adrenal medullary transplants into subarachnoid space of five palliative patients with intractable cancer pain [38](Table 1), amongst them there was 80% response rate with significant reduction of pain and demand for opioids when they were followed up to 1 year. Two other clinical studies from France using adrenal medullary transplants, one as a pilot with two patients having chronic pain [39](Table 1), the other a Phase II study with 15 patients diagnosed with intractable cancer pain [40], reported similar clinical improvement plus increase in CSF met-enkephalin levels. Albeit such promising results, the use of adrenal medullary tissues carries three limitations: (i) the minimal effective dose is still unclear and the clinical response did not seem to be dose-dependent; (ii) the time limit of the donor medullary tissue to maintain its viability and antinociceptive potency is uncertain [41]; (iii) donor shortage in clinical situation. To circumvent these difficulties, isolated bovine chromaffin cells encapsulated by semipermeable polymer membranes were successfully employed in sheep recipients as a functional xenogeneic transplant with a dose-related response [42]. This led to the development of a prototype implant which was pilot-tested in seven patients with severe non-cancer type of chronic pain [43](Table 1), with data showing acceptable levels of safety and device retrievability without the need of immunosuppression.

**Table 1:** Synopsis of clinical studies using cellular therapies for chronic pain

Modality	Methods	Outcome	References
Primary adrenal medullary/chromaffin tissue	Cadaveric adrenal medullary transplants into 5 subjects with intractable cancer pain	80% response rate with reduced demand for opioid analgesia	[38]
	Allograft to lumbar in 2 subjects with chronic cancer pain	Clinical improvement with increase in CSF Met-enkephalin levels	[39]
	Encapsulated bovine chromaffin cells implanted as a device in subarachnoid space of 7 subjects with chronic pain	Reduction of morphine requirement from 30-100% within a period of 41-176 days post-implantation	[43]
	Phase II trial with allograft to CSF space in 15 subjects with cancer pain	Reduction of intra-thecal morphine dosage and increase in CSF Met-enkephalin levels	[44]

Bone marrow-MSC	Bone-marrow MSC co-cultured with autoimmune T-cells given to 2 human subjects with chronic SCI	Recovery of motor and sensory functions up to 8 spinal cord levels within 6 months	[45]
	Open label case-control study with 64 subjects (44 as trial and 20 as control) using monthly intrathecal autologous MSC transplant for 6 months	No significant differences found in terms of ASIA score, 55.8% of treated subjects developed neuropathic pain	[46]
	Three cycles of allogeneic MSC treated CD34 cells given over 14 months to a subject with incomplete SCI	Reduction of neuropathic pain by 70% and resumption of motor and sexual activities	[47]

Bone marrow transplant	Unmanipulated autologous bone marrow transplant to 20 subjects with complete SCI	Regime generally safe and feasible	[48]
	Phase I/II open label trial with 35 subjects having complete SCI receiving autologous bone marrow with GM-CSF	Clinical improvement in 30.4% of subjects with no complication of tumour or neuropathic pain formation	[49]
	Uncontrolled series in Ecuador with 52 subjects with SCI given bone marrow stem cells	Clinical improvements described	[50]
	Phase I/II study with 297 patients with SCI receiving single unmanipulated autologous bone marrow cells	Regime relatively safe with improvement in motor/sensory functions in 1/3 subjects	[51]
	Clinical pilot with 30 subjects with SCI receiving single dose of ex-vivo expanded bone marrow transplant	Clinical improvement in subjects with < 6 months injury, not sure if effects due to spontaneous recovery	[52]

Olfactory ensheathing cells (OEC)	Phase I/IIa study with 6 subjects with thoracic paraplegia receiving autologous OEC and followed up at 1 yr and 3 yrs	1 out of 6 subjects had mild clinical improvement	[53,54]
	Uncontrolled trial with 16 subjects receiving heterologous OEC from aborted foetuses	No improvement mentioned	[55]
	Pilot study with seven subjects (ASIA class A) having olfactory mucosa autografts (OMA) into spinal cord lesions, later escalated to a prospective study with 20 subjects	Feasible and safe procedure with improvement with ASIA scores, bladder sensations and sphincter functions, with additional radiological improvements in the prospective study	[56,57]
	Pilot study with 5 subjects with chronic SCI receiving OMA	No significant improvement, development of syrinx in one subject and myelomalacia in other 4	[58]

Schwann cells	Pilot study with 4 subjects receiving autologous transplant from sural nerve cultures	Overall no adverse effects with improvement in only one subject	[59]

Anti-TNF-α	One report of current usage of etanercept in one subject with accident of T7 cord transection	Significant reduction of inflammation and motor improvement	[60]

Anti-Nogo-A	Phase I study with anti-Nogo-A given to > 50 subjects within 14 days of SCI	Still under evaluation	[61]

### Using immortalised cell lines

Details of immortalising cell lines prepared under laboratory conditions are beyond the scope of this paper. Suffice to say in the research of neuropathic pain, Eaton *et al. *[62,63] made significant contribution in immortalising two main cell lines which helped advance the study of cellular therapy for pain. One of them is a neural cell line RN33B, derived from E13 brainstem raphe and immortalised with SV40 temperature-sensitive allele of large T antigen (tsTag), which are then conditioned to proliferate at 33°C and stop proliferation at 39°C. RN33B can further be transfected with cDNA either for synthesizing galanin [64], BDNF [65] or GABA [62], either of which when transplanted in the lumbar subarachnoid space of rats with experimental chronic constricting injury (CCI), demonstrated significant reduction of neuropathic pain [62,64,65]. The second cell line is bovine chromaffin cell line, where the immortalised cells possess immuno-reactivities for met-enkephalin, galanin GABA and 5-HT without further gene transfection with minimal in vivo tumorigenicity [63]. Despite the theoretical advantage of its unlimited supply, these immortalised cell lines still carry oncogenic potential and hence none of them has yet been approved for clinical trial as a cellular therapy for spinal cord injury without demonstrating a fool-proof reassurance of dis-immortalisation. Also, there is as yet a reliable method of controlling the immortalised cells to deliver the specific dose of neurotrophins for the desired effect on neuropathic pain. That is especially relevant when the secreted neurotrophins produce antagonistic effects on pain at different concentrations: an excellent example will be BDNF, where Miki *et al. *[44](Table 1) demonstrated that systemic infusion of BDNF to rats with ligated spinal nerves relieved mechanical neuropathic pain at a lower concentration of 1 μg/h and, paradoxically, enhanced the pain response when given at a higher dose of 20 μg/h.

### Using other engineered cell lines

Xu *et al. *[66] transfected and immortalised astrocytes with cDNA carrying the human preproenkephalin gene (hPPE) which was combined with the tetracycline-controlled (Tet-on) expression system, and these astrocytes were implanted into the subarachnoid space of rats with CCI. The group documented significant rise of spinal enkephalin in these rats as regulated by doxycycline administration in a dose-dependent fashion, with alleviation of thermal and mechanical hyperalgesia. In a recent experiment [67], the same group applied similar protocols using preprogalanin cDNA (without tetracycline-controlled expression system) and found increased spinal galanin with overall reduction of thermal hyperalgesia and mechanical allodynia. In a different note, Liu *et al. *[68] in 2004 engineered a replication-incompetent herpes simplex virus (HSV) vector expressing one isoform of the human glutamic acid decarboxylase (GAD) and transfected rats with T13 spinal cord hemisection via subcutaneous inoculation. The recipient rats exhibited less neuropathic pain presumably due to increased levels of GABA at the dorsal root ganglion (DRG). Lee *et al. *[69] conducted a similar study but in addition, explored the effects of another strain of non-replicating HSV vector expressing proenkephalin. He found the reduction in pain behaviour was less significant with the proenkephalin-expressing vectors. In 2009, Miyazato *et al. *[70] injected GAD expressing HSV-vectors into the bladder walls of rats with SCI and found alleviation of detrusor overactivity, supporting the hypothesis that the GAD gene therapy enhanced GABA-mediated suppression of neuropathic signals. Encouraged by these experimental findings, Wolfe *et al. *[71] embarked upon a Phase I single-centre, open-label, dose-escalating trial using a clinical grade of replication-incompetent HSV virus expressing human preproenkephalin gene (called NP2). Patients with intractable pain due to malignancy below the angle of the jaw were enrolled and NP2 virus was administered by innoculation. At time of writing, the trial is still in progress and results are pending.

### Using stem cells

Use of stem cells has phenomenally advanced regenerative medicine and equally has aroused enormous medico-legal controversies, especially regarding the use of embryonic stem cells derived from human beings. In the last decade, mesenchymal stem cells (MSC) have remained a major focus of stem cells research. MSC are found in the adult bone marrow with a mesodermal origin and are capable of differentiating into cells that constitute the blood, adipose tissue, connective tissues, the vascular and urogenital system [72]. In vitro, MSC can be expanded easily from a small amount of bone marrow aspirate with stable phenotype and genotype, and are easily transported in various methods and formulations from the bench to the bedside [73]. Moreover, MSC migrate to sites of tissue injury with extraordinary immunosuppressive properties and their ability to differentiate into neurons and astrocytes have been documented both in vitro and in vivo [74]. In addition, MSC can enhance synaptic transmission and promote neuronal network in mice model of neurodegeneration [75], making MSC a prime candidate for nervous system repair. In the context of neuropathic pain, direct injection of human mesenchymal stem cells (hMSC) into the cerebral ventricle of mice with spinal nerve injury (SNI) reduced formation of neuropathic pain [76]. So far, three clinical studies have been published regarding the use of bone marrow MSC for spinal cord injury. In 2006, basing on their previous findings that human MSC derived from adult bone marrow can trans-differentiate into neural stem cells when co-cultured with auto-reactive T-lymphocytes [77], Moviglia *et al. *[45](Table 1) reported clinical extension of spinal cord function in two patients when given these MSC pre-primed with anti-T cells autoserum and noted no apparent adverse effects. In 2009, Kishk *et al. *[46] (Table 1) conducted an open label case-control study with 64 patients who had SCI within an average of 3.6 years. 44 patients consented to monthly autologous MSC transplant for 6 months, which was given intrathecally. The 20 patients who refused therapy served as controls. All patients were evaluated for adverse effects and functional improvements 12 months after the therapy. Results were disappointing as no significant between-group improvements as per clinical measures were detectable, with additional adverse effects of spasticity in 9.3% and neuropathic pain in 55.8% of subjects who received therapy. In 2010, Ichim *et al. *[47](Table 1) gave three cycles of combined allogeneic MSC and expanded umbilical cord blood CD34 cells intrathecally over a period of 14 months to a patient with incomplete spinal cord injury. They reported significant reduction of neuropathic pain from an intermittent 10/10 to weekly 3/10 basing on the visual analogue scale (VAS). Other improvements in terms of muscle, bowel and sexual function were also noted without any adverse effects.

### Using bone marrow and bone marrow stem cell transplant

Instead of using the specific mesenchymal portion of bone marrow, several clinical studies have employed autologous whole bone marrow transplant or its stem cell abstract for treating spinal cord injury. In 2006, a case study from Czech republic [48](Table 1) recruited 20 patients with complete SCI and were given unmanipulated autologous bone marrow transplant intra-arterially 10 to 467 days post-injury. Results showed general level of safety with improvement in terms of sensory and motor functions mostly amongst the acutely injured group, and the authors cautioned that the observed benefits might be confounded by the natural recovery processes. Yoon *et al. *[49](Table 1) conducted a Phase I/II open label non-randomised study in 2007 with 35 patients diagnosed with complete spinal cord injury. They received autologous bone marrow cell transplant together with granulocyte macrophage-colony stimulating factor (GM-CSF) within 8 weeks of injury directly at the site of spinal lesion. No adverse effects of tumour, cysts or neuropathic pain was observed up to 10.4 months after injury, with improvement of clinical measures up to 30.4%. In 2008, Geffner *et al. *[50](Table 1) in Ecuador also described improvement in clinical measures with relatively minor adverse effects when bone marrow stem cells were given to an uncontrolled series of 52 patients with SCI via multiple routes of administration(spinal cord injection, spinal canal injection and intravenous). By far the largest Phase I/II study was conducted in 2009 by Kumar *et al. *[51](Table 1) in India. 297 patients who had SCI were enrolled and they received a single treatment of unmanipulated autologous mononuclear bone marrow cells transplantation via lumbar puncture. They were then followed for up to a mean of 20.4 months and approximately 1/3 of patients showed some form of sensory or motor improvements, which were dependent on the absolute number of CD34+ cells transplanted. The group concluded that such treatment was relatively safe without any serious adverse effects. However, not all SCI clinical studies using bone marrow transplants yielded convincing benefits. A pilot conducted by Pal *et al. *[52](Table 1) in 2009 recruited thirty patients with complete SCI within 6 months of injury. They received a single treatment of autologous ex-vivo expanded bone marrow transplant via lumbar puncture and showed no harmful effects but benefits were only apparent for patients with less than 6 months of injury. However, as most patients with acute SCI will recover spontaneously within 3 to 18 months regardless, it is necessary to have a large enough sample size to demonstrate validity and statistical significance for true benefits of any therapeutic intervention [78]. Hence, Pal *et al. *rightly questioned if their observed improvements were genuinely due to treatment itself.

## Other cell-related strategies for SCI with implications to neuropathic pain

### Olfactory ensheathing cells (OECs)

The olfactory mucosa is a fascinating anatomical area with incessant regenerative potential. It contains both multipotent progenitor cells and olfactory ensheathing cells (OECs), the former capable of differentiating into both neural and non-neural cells [79], and the latter capable of promoting axonal remyelination and regeneration after injury. It is of interest to note that OECs, though normally associated with axons of the first cranial nerve, do not myelinate the olfactory nerve per se. They only assume a myelinating prototype when transplanted to the vicinity of axons of larger diameter [80]. Research findings in the last decade have suggested that transplanting OECs into damaged spinal cord promotes axonal regeneration and remyelination, facilitating overall functional recovery of the spinal cord [81-83]. However, this view has been challenged when OEC graft transplanted to rats with rhizotomy failed to enable axonal regeneration beyond the dorsal root entry zone [84-86]. Moreover, controversy has been intense as to whether these regeneration-capable olfactory cells are OECs or in fact, Schwann cells. OECs resemble Schwann cells so closely in terms of biochemistry, microscopic morphology and molecular transcription that it is often impossible to distinguish between the two [80]. Basing on their earlier findings with genomic studies that calponin is a definitive phenotypic marker for OECs which is not shared by Schwann cells [87], Kawaja's group found that primary cultures of olfactory mucosa and bulb often contained a mixture of calponin-positive OECs and calponin-negative Schwann cells [88]. In other words, what is normally thought as "OECs culture" will be invariably contaminated by Schwann cells. Hence, the concept of OECs remyelinating damaged axons without the influence of Schwann cells might need revision. In a recent authoritative review, Kawaja *et al. *[89] exhaustively critiqued the technical strategies of obtaining and culturing OECs from olfactory mucosa or olfactory bulb of various animal species and humans, the different biomarkers used for identifying OECs, and offered a state-of-the-art opinion on the controversy of Schwann cell contamination amongst OECs. Thus said, experimental data have shed enough light for clinical trialing of OECs in patients with SCI. In 2004, Mackay-Sim *et al. *conducted a Phase I/IIa study using autologous transplantation of OECs in six patients with thoracic paraplegia with follow-up at one year [53] and three years [54](Table 1), and the group concluded that such procedure seemed to be safe with no consequences of iatrogenic neuropathic pain or tumour formation. However, only one out of the six subjects showed improvement in sensory function over three segments of the thorax. In 2006, Huang *et al. *[55](Table 1) followed 16 patients with SCI who received heterologous OECs transplants from aborted foetuses and they found no major adverse effects or pathology within 38 months of the procedure. There was, however, no mention of functional improvement or clinical symptoms. More favourable outcomes were reported by Lima *et al. *[56](Table 1) who conducted a pilot study with seven patients having olfactory mucosal autografts (OMA) directly transplanted into their spinal cord lesions. They exhibited good improvements in bladder sensation, anal sphincter function and overall paraplegic scores according to the American Spinal Injury Association (ASIA). There was also corresponding remyelination of the lesional sites as documented by spinal MRI scan. The same group hence proceeded to a prospective study in 2010 [57] (Table 1) using the same protocol with a larger sample size of 20 patients. They confirmed similar clinical benefits and radiological improvements as with their initial pilot study. However, such promising findings were not replicated in the five patients recruited by Chhabra *et al. *[58](Table 1), for which the authors attributed to the procedures involved. In addition, adverse effects of syrinx formation in one subject and extension of myelomalacia in four subjects were reported.

### Schwann cells

Discovered by Theodore Schwann (1810-1882), Schwann cells are a major component of the peripheral nervous system derived embryonically from the neural crest cells. Schwann cells grow in juxtaposition to axons and also myelinate them. Following axonal injury, Schwann cells de-differentiate into the non-myelinating phenotype and proliferate, secreting an array of growth modulators like collagen IV, laminin and fibronectin in the surrounding domain [90]. In experimental models of SCI, Schwann cells were found to be present in the regenerated areas [91-93], which helped to regenerate the axons with various neurotrophic factors like nerve growth factor (NGF)[94], brain-derived neurotrophic factor (BDNF)[95], Neutrotrophin-3 (NT-3)[96], glial derived growth factor (GDNF)[97] and pleiotrophin (PTN, HB-GAM) [98]. In particular, Schwann cells seem to exhibit distinct motor or sensory phenotypes as per immunoreactivity towards PTN which direct regenerating axons towards the specific phenotypes [99]. Recent improvement in cell harvesting and proliferation techniques have enabled human Schwann cells to be obtained in a sufficiently large and purified amount for reparative purpose of spinal cord injury. Saberi *et al. *[59](Table 1) studied the effects of human autologous Schwann cell transplant purified from autologous sural nerve culture in four patients with mid-throacic spinal cord injury. They reported lack of adverse effects overall with improvement in sensory and motor functions in only one patient, and MRI scanning failed to show any corresponding changes in white matter.

### Specific anti-cytokine treatment: the rise and fall of TNF-α

As mentioned above, SCI leads to a myriad of neuroinflammatory mediators which contribute to the pathogenesis of neuropathic pain. Amongst them, tumor necrosis factor-alpha (TNF-α) is one of the most extensively studied which can be detected promptly after experimental models of spinal cord injury [100]. First discovered in 1891 from a mixed extract of *Streptococcus pyogenes *and *Serratia marcescens *bacteria [101] and later characterised with tumor-regression activity [102], TNF-α belongs to a superfamily of ligand/receptor proteins called the tumor necrosis factor/tumor necrosis factor receptor superfamily proteins (TNF/TNFR SFP). TNF-α possess a trimeric symmetry with a structural motif called the TNF homology domain (THD), which is shared with all other members of the TNF proteins. This THD binds to the cysteine-rich domains (CRDs) of the TNF receptors (TNFRs), and variations of these CRDs lead to heterogeneity of the TNFRs [103]. TNFRs are either constitutively expressed (TNFR1, p55-R) or inducible (TNFR2, p75-R) [104]. The inducible TNFR2 forms the basic architecture of etanercept, an FDA approved drug for treating severe rheumatoid arthritis and plaque psoriasis. In the context of neuropathic pain, using the CCI model in rats, TNF-α is detectable at the injury site in a temporal up-regulation [105-107], located mainly in both the macrophages [108] and the Schwann cells [109,110]. Similarly, there is local up-regulation of both TNFR1 and TNFR2 as the injured neurons undergo Wallerian degeneration, albeit at a differential rate [111]. Further upstream, there is enhanced TNF immunoreactivity in the dorsal root ganglion (DRG) of both the injured and spared ipsilateral adjacent afferents [112], plus the contralateral uninjured counterparts [113], which can only be partly explained by retrograde axonal transport [114]. There is also a corresponding up-regulation of TNFR1 and TNFR2 in both the nerve and the DRG [115], with a temporal pattern of an increase of TNF mRNA expression, first in the sciatic nerve, and then in the DRG [116]. Finally, glial TNF-α is thought to play a role in mediating the central mechanisms of neuropathic pain. Using classic CCI models in rats, increased levels of TNF-α are found in the hippocampus [117,118], locus coeruleus [118,119] and the red nucleus [120] of the brain. Albeit the ubiquity of TNF-α following experimental models of spinal cord injury, randomised controlled clinical trials of infliximab (antibody to TNF-α) and etanercept (recombinant TNFR2) have not demonstrated benefits for patients with discogenic sciatica [121-124], which thwarted further research of anti-TNF-α treatment for other types of neuropathic pain. In comparison, clinical trial regarding the use of anti-TNF-α for spinal cord injury or related neuropathic pain is lacking, with only one case study reporting significant motor improvement and reduction of inflammation at the injured cord area of a patient who incidentally received etanercept treatment for ankylosing spondylitis shortly before a T7-cord transection accident [60](Table 1).

### Disinhibiting axonal regrowth: no go to nogo

The spinal cord attempts to self-repair after any injury, which often ends in failure due to a combination of glial scar and growth inhibitors associated with myelin. Glial scar is formed by a congregation of meningeal fibroblasts, activated astrocytes, microglia and oligodendrocytes which on one hand, physically barricade the regenerating axons and on the other hand, secret an array of chondroitin sulphate proteoglycans (CSPGs) which chemically deter neurite outgrowths [125]. Presumably from the initial surge of Schwann cell activity to repair and myelinate the damaged axons [126], myelin-associated growth inhibitors like Nogo-A, myelin-associated glycoprotein (MAG) and oligodendrocyte-myelin glycoprotein (OMG)[127] rapidly dominate the area and prevents further axonal growth and regeneration. Experiments with animal transgenic mutants deficient in these myelin-associated inhibitors have demonstrated better axonal growth and locomotory functions after spinal cord injury [128,129], which fosters the idea of antagonising these myelin-associated inhibitors to enable axonal re-growth and hence spinal cord regeneration. In 2010, a Phase I clinical trial of anti-Nogo-A in patients with acute spinal cord injury has been embarked by Zorner *et al. *[61](Table 1).

### Against cell cycle activation

From animal experiments, there is evidence that cell cycling is activated during SCI which leads to neuronal cell death [130,131], oligodendrocytes loss [131], inflammation [132,133], tissue scaring [134] and astrogliosis [132]. Increased cell cycling is seen by enhanced production of cycle proteins like cyclins [135], cyclin-activated kinases [136,137] and inhibitors of cyclin-dependent kinases [138]. It has been shown that cell cycle inhibitors are capable of reducing axonal damage and lead to functional recovery [131,132,134] in animal models of spinal cord injury. Other inhibitory molecules that affect axonal regeneration in spinal cord injury include the Wnts molecules [139], semophorin 3(SEMA3)[139] and the RhoA signalling pathway [140]. Despite the state of accumulated knowledge, no clinical trials have been approved or in progress to test the efficacy of these cell cycle inhibitors on patients with SCI.

### Neurotrophins: a friend or foe for spinal cord injury?

Neurotrophins refer to the class of growth factors in the CNS that promote growth, maintenance and survival of neurons and synapses. They comprise of NGF, BDNF, NT-3 and NT-4/5 [40]. In rats and primates, the levels of neurotrophins peak initially in the embryonic stage where neurons and synapses are produced in abundance for matching and paring, after which the levels generally decline when inappropriate neurons and synapses are eliminated towards the adult neuronal profile [141,142]. Following acute hemisection of the spinal cord in Rhesus monkeys, the levels of NGF, BDNF and NT-3 decreased sharply from day 3 to Day 7 and increased persistently up to Day 30 [143], consistent with the attempt of intrinsic neuronal repair. Coupled with other experimental data showing that exogenously applied neurotrophins promote regeneration of various neuronal populations after various periods of spinal cord injury [144-146] even up to a period of 15 months [147], neurotrophins would be a promising therapy for spinal cord repair in humans. However, caution is needed in several areas when translating the positive findings from pre-clinical studies in rodents to clinical consideration: (i) there are species-specific differences in neuro-plasticity which necessitate regeneration of the corticospinal tracts in primates when restoring sensori-motor activities during spinal cord injury, but such pre-requisite is not shared by rodents [148]; (ii) the role and specificity of neurotrophins involved in promoting axonal regeneration and preventing corticospinal neuronal atrophy in spinal cord repair maybe different [149]; (iii) neurotrophins as therapy for CNS regeneration can lead to undesirable effects, e.g., exogenous administration of BDNF in experimental models can contribute to spinal nerve injury-induced neuropathic pain by activation of microglia [150] and astrocytes [151], whilst intra-cerebroventricular infusion of NGF leads to weight loss and neuropathic pain in one clinical pilot [152].

## Hurdles of cellular therapies in human

Despite the promising prospect from various modalities as described above, there are a few hurdles to be surmounted before cellular therapies can be channelled towards large-scale clinical trials and eventual bedside use. For convenience of discussion, we shall choose stem cell therapy to illustrate such challenges.

### Cells generation and homogeneity

There is as yet a standardised and efficient protocol to produce a specific type of stem cells in quantities large enough for clinical therapy. The combined protocol of feeder cells, growth factors and genetic modulation has been the traditional method for expanding the colonies of hESC [153] with average yield of < 20% [140], which in defined conditions can be enhanced to 95% with small molecule induction using retinoic acid [154]. In reality, it remains a technical challenge to conform stem cell differentiation to a particular phenotype, although a novel approach with nanofiber-scaffold drug release technology has recently been reported to be successful in committing MSC towards a neural differentiation [155]. Genome wide analysis has been used to monitor the quality and differentiation of hESC lines regarding the dynamics of "stemness genes"[156] and possible feeder contaminations [157]. It would be beneficial if transcriptomic and proteomic datasets are readily available for neuroscientists studying hECS to a degree as for studying OEC [158] and MSC [159].

### Cell dosing and delivery

Dosing of stem cells in terms of upper limit and frequency of administration remains controversial. Intuitively one would expect an incremental response with dose escalations but available data is limited. Recent study by Usvald *et al. *[160] demonstrated an optimum dosing regimen for intra-parenchymal injection of human spinal stem cells into minipigs spinal cord for the best neuronal repopulation. Other studies have suggested that intra-thecal and intra-venous delivery of stem cells were less efficacious than direct injection into the spinal cord tissue, albeit the danger of further damage to the lesion [161,162]. Vaquero *et al. *[163] also showed that intra-lesional injection of stem cells in rats with SCI produced better outcome than intravenous administration. Thus said, it is still a challenge to target the stem cells within cellular precision. Recent study by Wu *et al. *[164] proposed the use of fibrin glue as a vehicle for delivering MSC to injured neural tissues.

### Cell tracking and outcome measure

It would be ideal to track the stem cells after being administered to determine their distribution, location, quantity, viability and final differentiation for both research and clinical purposes. Non-invasive strategies include (i) direct labelling of target cells with paramagnetic contrast agents and tracking them with functional MRI, either using gadolinium [165,166], supermagnetic iron oxide particles [167,168], or ^19^F isotope [169]; (ii) direct labelling with traditional fluorochromes like PKH26 [170] or quantum dots using cadmium nanocrystals [171,172]; (iii) internal labelling using transfected enhanced green fluorescence protein (eGFP) and firefly luciferase (fLuc) reporter genes either via the bioluminescence mechanism [173,174]. Each of these cell tracking methods suffer from drawbacks: paramagnetic contrast uptake can be limited and MRI signals maybe weak (except using the supermagnetic iron oxide particles); traditional fluorochromes are prone to bleaching whilst cadmium in quantum dots crystals are toxic to cells; finally, bioluminescence imaging maybe be limited by the low tissue penetrance [175].

## Conclusions and future directions

Spinal cord injury is a devastating condition in humans leading to significant disability with immense loss of quality of life and economic output. At time of writing, there is no satisfactory clinical cure and overall prognosis is poor. In the last two decades, experimental data using cellular or cell-related therapies have opened up exciting therapeutic possibilities. Various clinical studies using cellular therapies for spinal cord injury have been discussed above and are summarised in Table 1 for ease of reference. For spinal cord regeneration, stem cell transplantation still holds the best future and amongst them, hESC [176] and OEC [177] are currently the prime candidates. However, with the abrupt withdrawal of Geron from the GRNOPC1 Phase I clinical trial, the actual immaturity of hESC research in spinal cord injuries and its vulnerability to financial considerations is well illustrated [178]. As regards to combating neuropathic pain related to spinal cord injury, cellular or cell-related therapies are rapidly gathering momentum which aim to achieve analgesia from different perspectives: preventing neuronal damage due to inflammation, cell cycling or dysfunctional regeneration; installing biological mini-pumps using adrenal medullary chromaffin tissues, engineered cell lines or astrocytes and finally, regulating the internal milieu using transplant of bone marrow of bone marrow mesenchymal stem cells.

## Abbreviations

5-HT: 5-hydroxytrytamine; AMPA: α-amino-3-hydroxy-5-methyl-4-isoxazolepropionic acid; ASIA: American Spinal Injury Association; BDNF: Brian derived neurotrophic factor; CCI: Chronic constricting injury; CCL2: Chemokine (C-C motif) ligand 2; CSF: Cerebrospinal fluid; CSPG: Chondroitin sulphate proteoglycans; CX3CL1: Chemokine (C-X3-C motif) ligand 1; DRG: Dorsal root ganglion; GABA: γ-aminobutyric acid; GAD: Glutamic acid decarboxylase; GDNF: Glial derived growth factor; GM-CSF: Granulocyte macrophage-colony stimulating factor; HB-GAM: Heparin-binding growth-associated molecule; HSV: Herpes simplex virus; IL-1β: Interleukin-1 beta; IL-6: Interleukin-6; MAG: Myelin-associated glycoprotein; MRI: Magnetic resonance imaging; MSC: Mesenchymal stem cells; NGF: Nerve growth factor; NMDA: N-Methyl-D-aspartic acid; NT-3: Neurotrophin-3; NT-4/5: Neurotrophin-4/5; OEC: Olfactory ensheathing cells; OMA: Olfactory mucosal autografts; OMG: Oligodendrocyte-myelin glycoprotein; PAG: Periaqueductal gray; PTN: Pleiotrophin; SCI: Spinal cord injury; SNI: Spinal nerve injury; THD: Tumor necrosis factor homology domain; TNF-α: Tumor necrosis factor alpha; TNFR: Tumor necrosis factor receptor; VAS: Visual analogue scale.

## Competing interests

The author declares that they have no competing interests.

## Authors' contributions

LL performed the review and wrote the manuscript.
